# The Emerging Roles of Heparan Sulfate 3-*O*-Sulfotransferases in Cancer

**DOI:** 10.3389/fonc.2019.00507

**Published:** 2019-06-12

**Authors:** Agnès Denys, Fabrice Allain

**Affiliations:** Univ. Lille, CNRS, UMR 8576 - UGSF - Unité de Glycobiologie Structurale et Fonctionnelle, Lille, France

**Keywords:** heparan sulfate, sulfotransferase, cancer, epigenetic regulation, immune escape

## Abstract

Alteration in the expression of heparan sulfate (HS)-modifying enzymes has been frequently observed in cancer. Consequently, dysregulation of the HS biosynthetic machinery results in dramatic changes in the HS structure, thereby impacting a range of pivotal cellular processes involved in tumorigenesis and cancer progression including proliferation, migration, apoptosis, and immune escape. HS 3-*O*-sulfotransferases (HS3STs) catalyse the maturation step of glucosaminyl 3-*O*-sulfation within HS chains. Although seven HS3ST isozymes have been described in human, 3-*O*-sulfation is a rare modification and only a few biological processes have been described to be influenced by 3-*O*-sulfated HS. An aberrant expression of HS3STs has been reported in a variety of cancers. Thus, it was suggested that changes in the expression of these enzymes as a result of tumorigenesis or tumor growth may critically influence cancer cell behavior. In accordance with this assumption, a number of studies have documented the epigenetic repression of HS3ST2 and HS3ST3A in many cancers. However, the situation is not so clear, and there is accumulating evidence that HS3ST2, HS3ST3A, HS3ST3B, and HS3ST4 may also act as tumor-promoting enzymes in a number of cancer cells depending on their phenotypes and molecular signatures. In this mini-review, we focus on the recent insights regarding the abnormal expression of HS3STs in cancer and discuss the functional consequences on tumor cell behavior. In term of clinical outcome, further investigations are needed to explore the potential value of HS3STs and/or their 3-*O*-sulfated products as targets for therapeutic strategies in cancer treatment.

## Introduction

Heparan sulfate (HS) is an anionic and linear polysaccharide, which is covalently attached to core proteins to form HS proteoglycans (HSPG). These molecules are present within the extracellular matrix (ECM) and at the surface of virtually all cells. While the core protein primarily determines the localization of HSPG, HS chains are involved in the binding of a large number of proteins, including growth factors, cytokines, proteases, lipoproteins, and ECM components. HS-protein interactions have multiple effects ranging from simple immobilization to protection against degradation, conformational change, stabilization of receptor-ligand complexes, or protein oligomerization (1–5). Via these interactions, HS does not only regulate physiological processes, such as in embryogenesis, angiogenesis, blood coagulation and inflammation, but are also implicated in many pathologies, including cancer, infectious diseases, and neurodegenerative disorders (6–10).

**Figure 1:**
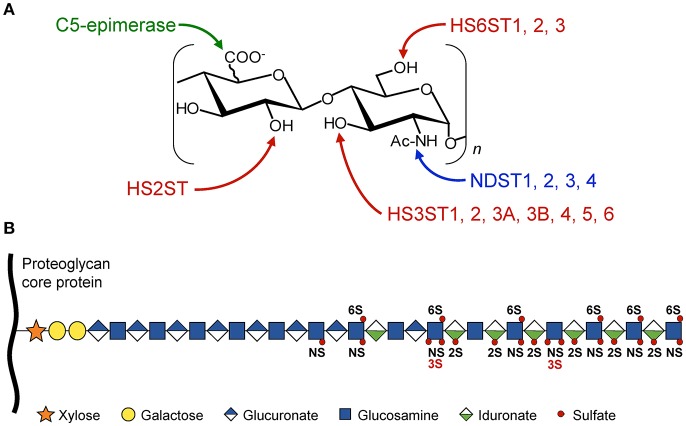
Schematic representation of HS modifications. The HS polysaccharide is linked by the tetrasaccharide linker [Xyl-Gal-Gal-GlcUA] to a specific serine residue within the HSPG core protein. Elongation of the chain is achieved through the alternative addition of GlcUA and GlcNac residues by the polymerases EXT1/EXT2. **(A)** The disaccharide units [GlcUA-GlcNAc] are modified by the actions of sulfotransferases and epimerase. The sites of modification by *N*-deacetylases/*N*-sulfotransferases (NDST), C5-epimerase, HS 2-*O*-sulfotransferase (HS2ST), HS 6-*O*-sulfotransferases (HS6STs), and HS 3-*O*-sulfotransferases (HS3STs) are indicated. **(B)** HS modifications do not go to completion, resulting in domains with high, intermediate, and low levels of sulfation, enabling the generation of many HS structures and potential ligand-binding sites. NS, *N*-sulfo group on GlcN residue; 2S, 2-*O*-sulfo group on uronic acid residue; 3S and 6S, 3-*O* and 6-*O*-sulfo groups on GlcN residue.

Structural determinants in HS are derived from enzymatic modifications of the glycan backbone, which is formed by polymerization of the repeat unit consisting of D-glucuronic acid (GlcUA) and *N*-acetylated D-glucosamine (GlcNAc). In the classical model of biosynthesis, the native polysaccharide is first subject to partial *N*-deacetylation/*N*-sulfation of GlcNAc residues. This modification provides the substrate for next modifications, including epimerization of some GlcUA into L-iduronic acid (IdoUA), 2-*O*-sulfation of uronic acids (mainly IdoUA), and 6-*O* and/or 3-*O*-sulfations of GlcN residues (Figure 1). HS-protein interactions are primarily driven by complementarity between positively charged amino acid residues in the ligand and sulfate groups in the HS sequence. However, protein binding to HS does not only rely on the overall degree of sulfation. Instead, a concept has emerged whereby optimal binding depends on the spatial arrangement of sulfate groups in given HS sequences (2, 4, 5, 11, 12).

HS3STs represent the largest family of HS-modifying enzymes, and yet the reaction of 3-*O*-sulfation is the rarest maturation step, when compared to other sulfations. Seven HS3STs have been characterized in human, for which the expression is dependent on cell type and tissue environment (Table 1). HS3ST-mediated 3-*O*-sulfation leads to at least two distinct forms of 3-*O*-sulfated motifs. HS3ST1 and HS3ST5 participate in the generation of anticoagulant-active HS/heparin sequences for antithrombin-III, while HS3ST2, HS3ST3A, HS3ST3B, HS3ST4, and HS3ST6 were described to provide the HS-binding motifs for the glycoprotein gD of herpes simplex virus-1 (HSV-1) (13, 14, 33–41). To date, only a few ligands are known to selectively interact with 3-*O*-sulfated motifs, whereas hundreds of HS-binding proteins have been identified. Consequently, little is known concerning the functions of 3-*O*-sulfated HS in biological processes, apart from their roles in anticoagulant properties of HS/heparin and entry of HSV-1 into host cells (12, 42, 43).

**Table 1:** Tissue expression of human HS3STs.

**Enzyme**	**Expression in normal tissues**	**Potential role in cancer**
HS3ST1	cerebellum (high), spleen (high), cerebral cortex, kidney, lung, stomach, small intestine, colon, testis, liver, heart, pancreas, placenta (13, 14)	/
HS3ST2	cerebral cortex (high), cerebellum, placenta, spleen, lung, stomach, small intestine, colon, testis (13, 14)	anti-oncogenic (15–23) pro-tumoral (24, 25)
HS3ST3A	liver, placenta, spleen, stomach, small intestine, colon, testis, heart, lung, kidney, pancreas (13, 14)	anti-oncogenic (26, 27); pro-tumoral (27)
HS3ST3B	liver (high), placenta (high), spleen (high), stomach, small intestine, colon, testis, skeletal muscle, heart, lung, kidney, pancreas (13, 14)	pro-tumoral (25, 28–31)
HS3ST4	cerebral cortex (high), cerebellum, stomach, spleen, testis (13, 14)	pro-tumoral (25, 32)
HS3ST5	skeletal muscle (high), placenta, cerebral cortex, cerebellum, small intestine, colon (13, 33, 34)	/
HS3ST6	liver, kidney (35)	/

Expression of the genes encoding HS-modifying enzymes is frequently dysregulated in cancer and other diseases (42, 44, 45). An aberrant expression of HS3STs has been reported in various cancers, suggesting that these enzymes and their 3-*O*-sulfated products may be involved in tumorigenesis and cancer progression. However, these reports reveal either anti-oncogenic or tumor-promoting effects (Table 1), and the mechanisms and consequences of HS3ST dysregulation in cancer still remain obscure (15–23, 26–28, 32).

## Anti-oncogenic Properties of HS3STs

In cancer cells, hypermethylation of CpG islands in gene promoters has been associated with the loss of expression of some susceptible genes, including tumor suppressor genes, and genes encoding products involved in DNA repair and apoptosis (44, 46, 47). In the attempt to identify novel silenced genes in breast cancer, Miyamoto et al. (22) found that the 5' region of the *HS3ST2* gene was hypermethylated in tumor tissue but not in surrounding non-cancerous tissue. As a consequence, the expression level of HS3ST2 was markedly reduced in the cancer sample compared with the matched normal counterpart. Then, they demonstrated that HS3ST2 was not expressed in cell lines representative of the different molecular breast cancer subgroups (48). Reversing methylation restored the expression of the enzyme, confirming the silencing effect of gene methylation. Moreover, *HS3ST2* gene hypermethylation was detected in the majority of primary breast cancer samples analysed, and also in human colon, lung and pancreatic cancers (22). Following this work, many clinical studies have been published examining the relationships between aberrant methylation of the *HS3ST2* gene and tumorigenesis. Hypermethylation was found at high frequency in gastric, breast, colorectal, prostate and cervix cancers, as well as in hematological neoplasms (15–23). In breast and cervix, hypermethylation of the *HS3ST2* gene occurs early during malignant transformation, suggesting a correlation between HS3ST2 silencing and progression of the disease (16, 23). Hwang et al. (18) demonstrated that the exogenous re-expression of HS3ST2 was efficient to inhibit cell migration, invasion and proliferation in various lung cancer cell lines. However, they found that the tumor size was not significantly different between patients with *HS3ST2* gene hypermethylation and those without, in spite of the anti-proliferative property of HS3ST2 observed *in vitro*. Hence, they emphasized the need of further investigations to validate HS3ST2 silencing as a prognostic/predictive biomarker (18).

Besides HS3ST2, an analysis of the methylation status of other genes encoding HS sulfotransferases in chondrosarcoma showed hypermethylation in proximal regions of the *HS3ST1* and *HS3ST3A1* genes. Exposure to a demethylating agent restored their expression, confirming that aberrant methylation had affected their transcription. Moreover, re-expression of HS3ST3A reduced the proliferative and migratory properties of chondrosarcoma cells, suggesting that silencing of this enzyme may have contributed to tumor cell growth and invasiveness (26). In the following study, Mao et al. (27) demonstrated that the *HS3ST3A1* gene is epigenetically repressed in breast cancer cell lines representative of the different molecular subgroups, except in the human epidermal growth factor receptor 2-positive (HER2+) cell lines. Re-expression of the enzyme in luminal A-type MCF-7 and triple negative MDA-MB-231 cell lines reduced cell proliferation *in vitro* and tumor growth in xenografted mice. Thus, the authors hypothesized that modification in HS structure may have hindered the interactions of growth factors with signalling receptors (27).

## Tumor-promoting Activities of HS3STs

Albeit that epigenetic repression of the *HS3ST2* gene was related to progression of many cancers, Vijaya Kumar et al. (24) reported that its re-expression in MDA-MB-231 cells led to an increase in cell viability and invasion. Likewise, we reported that MDA-MB-231 cells carrying HS3ST2 expression displayed a significant increase in proliferation and survival (25). The pro-invasive phenotype was however not observed in the MCF-7 cell line (24), suggesting that the consequence of HS3ST expression could be different depending on the breast cancer phenotype. In line with this assumption, Mao et al. (27) described that HS3ST3A expression enhanced proliferation and survival of HER2+ SKBR3 cells, but not in MCF-7 cells. However, these authors reported that HS3ST3A was also anti-proliferative in MDA-MB-231 cells, meaning that this isozyme produced opposite effect than the one promoted by HS3ST2 in the same cell line (24, 25). Although intriguing, we also found that overexpression of HS3ST3A did not have any effects on the proliferation of MDA-MB-231 cells. In contrast, forced expression of HS3ST3B and HS3ST4 had the same functional impact as observed in the case of HS3ST2 (25). These results suggest that the impact of HS3ST expression in breast cancer cells could be also dependent on the type of isozyme. One explanation may be that each HS3ST exhibits subtle differences in their substrate requirement. On this assumption, HS3ST3A may have a restricted substrate specificity, making it incapable of synthesizing 3-*O*-sulfated HS with a tumor-promoting property in MDA-MB-231 cells. Conversely, HS3ST2, HS3ST3B, and HS3ST4 may exhibit a broader selectivity or share, at least in part, some common acceptors.

In pancreatic cancer cells, high level expression of HS3ST3B was reported to induce epithelial-mesenchymal transition (EMT) and to enhance cell invasiveness *in vitro*. Moreover, HS3ST3B overexpression was associated with an increased angiogenesis in graft-bearing mice, supporting the idea that HS3ST3B could favor pancreatic cancer progression (29). In the continuity of this study, Zhang et al. (30) reported that high expression of HS3ST3B in U937 leukemia cells enhanced cell proliferation and survival, while its silencing had opposite effects. The advantage given by HS3ST3B was related to an increase in the production of vascular endothelial growth factor (VEGF) and activation of downstream signalling pathways. Next, the authors demonstrated that conditioned medium of HS3ST3B-expressing U937 cells had a promoting effect on angiogenesis, which was dependent on the secretion of VEGF. Finally, they confirmed that HS3ST3B effectively promoted leukemia cell proliferation and VEGF-dependent angiogenesis in xenografted mice (30). Most recently, a clinical study conducted in a cohort of lung cancer patients uncovered that HS3ST3B expression was upregulated in tumor biopsies compared to that in matched normal tissues (28). A high level expression of the enzyme was also observed in NSCLC cell lines. Silencing its expression reversed the mesenchymal phenotype, meaning that HS3ST3B is involved in the regulation of EMT in lung cancer cells in the same way as in pancreatic cells (28, 29). High expression level of TRF2 (telomere repeat binding factor 2), a protein normally involved in telomere protection, has been observed in various human cancers. Interestingly, the *HS3ST4* gene was identified as a transcriptional target of TRF2, and increasing TRF2 level led to an up-regulation of *HS3ST4* gene expression. Moreover, exogenous expression of either TRF2 or HS3ST4 in various tumor cell lines similarly resulted in increased tumor growth in xenografted mice, which suggests that the expression of this enzyme may be part of a pro-oncogenic pathway (32).

## HS3STs and Modulation of Signalling Pathways

Consistent with a pro-invasive phenotype, Erk1/2 and β-catenin signalling was upregulated in HS3ST2-expressing cells in an HS-dependent manner (24). As a consequence, the expression of several target genes involved in cancer cell invasiveness and survival was increased. High level expression of HS3ST3B in U937 leukemia cells was associated with activation of Notch-1, Erk1/2 and Akt signalling (30), and more recently, the tumor-promoting effects of HS3ST2, HS3ST3B, and HS3ST4 were related to sustained activation of Src, Akt, and NF-κB, and up-regulation of the anti-apoptotic proteins survivin and XIAP (25). Importantly, all these signalling molecules have been well described to play a critical role in promoting tumor growth and resistance to apoptosis (49, 50).

Most of the studies conducted with cancer cell lines reported that HS3ST overexpression resulted in an increase in the level of 3-*O*-sulfated motifs (24, 25, 27). Consequently, 3-*O*-sulfation may have influenced ligand binding to cell surface HS, leading to an alteration of diverse signalling processes. Whether 3-*O*-sulfation can modulate ligand-receptor interactions was however unknown, until neuropilin-1 (Nrp1) was described as a preferential ligand for 3-*O*-sulfated HS (43). Initially described as a co-receptor for VEGFs and class 3 semaphorins in endothelial cells and neurons, there is now evidence that Nrp1 is also expressed in a number of cancer cells, wherein it regulates cell growth, migration, invasion, and immune escape, by interacting with a broad spectrum of growth factors (51–53). Importantly, HS was reported to contribute to formation of a high-affinity complex incorporating Nrp1, VEGF, and cognate signalling receptors (54, 55). Zhang et al. (30) described that the tumor-promoting effect of HS3ST3B in leukemia cells was dependent on an autocrine activation of VEGF-dependent signalling pathways. Thus, it may be suggested that 3-*O*-sulfation of HS has improved interplay between Nrp1, VEGF, and its receptors. Besides VEGF, transforming growth factor (TGF)-β has been also identified as a ligand of Nrp1. Interestingly, HS3ST3B was described as a regulator of TGF-β-mediated EMT in NSCLC cells (28). Though not mentioned in the study, these findings suggest a possible participation of Nrp1 and 3-*O*-sulfated HS in the response induced by TGF-β. Along the same lines, we demonstrated that silencing of Nrp1 in MDA-MB-231 cells reversed the advantage given by HS3ST3B (31). Hence, these findings raise the possibility that the tumor-promoting properties of HS3ST3B could be dependent on the formation of signalling complexes containing Nrp1.

Besides the roles attributed to HS moieties, HSPG core proteins have binding properties that engage them in specific interactions with proteins involved in signalling and cytoskeleton organization (1, 3). A number of studies have reported that the expression of HSPG is dysregulated in many cancers, thus altering key biological processes involved in cell proliferation and survival (9). However, there is no evidence that changes in the expression of a core protein can alter the sulfation patterns within HS chains (56). Recently, Corti et al. (57) reported that HS chains of syndecan-2 contained higher levels of 6-*O* and 3-*O* sulfations, which was related to an increase in formation of a signalling complex between syndecan-2, VEGF and its receptor in endothelial cells. These last findings demonstrate the existence of a regulatory mechanism wherein a core protein determines the sulfation pattern of its own HS chains. High level expression of syndecan-2 has been observed in many cancers (9). This suggests that such a regulatory mechanism of HS sulfation may also occur in cancer cells. This could lead to the appearance of HSPG with specific HS chains, thereby enhancing the binding and functions of certain HS ligands. This assumption deserves additional works to identify the HSPG and their relevant ligands that interact with 3-*O*-sulfated HS in cancer cells.

## HS3STs and Escape to Immune Surveillance

A body of evidence has accumulated over the past two decades indicating that HS3ST2 is epigenetically silenced in a wide range of cancers and tumor cell lines (15–23). However, the authors did not address the possibility that another HS3ST could be expressed in place of HS3ST2. This assumption is supported by clinical studies showing that HS3ST3A and HS3ST3B were highly expressed in biopsies from patients with HER2+ breast cancer (27) and lung cancer biopsies (28), respectively. These observations suggest that HS3STs can compensate each other for loss of their expression depending on the molecular signature and tissue environment of cancer cells.

During cancer progression, developing tumor cells are exposed to pro-inflammatory mediators that enhance immune anti-tumoral response. In order to evade this immune pressure, tumor cells can change their intrinsic features, thereby resulting in the emergence of cellular variants with increased activation of pro-oncogenic pathways, and less immunogenic phenotype (58). It is of note that upregulation of the expression of HS3ST3B has been observed in many cell types exposed to inflammatory stimuli (28, 59–62). On the other hand, a progressive upregulation of TRF2 was observed during progression of colon cancer. Increased expression of TRF2 was associated with an abnormal expression of HS3ST4, which in turn led to inhibition of NK cell activation and recruitment. The same effects were observed with cancer cells carrying an exogenous expression of HS3ST4, suggesting that the isozyme may be involved in a mechanism of immune escape (32). In line with these findings, we reported that HS3ST-transfected MDA-MB-231 cells were more resistant to apoptosis induced by death receptors ligands or NK cells *in vitro* (25). The functions of certain NK cell receptors can be modulated through interactions in cis with HS on NK cells themselves or in trans with HS on target cells. Disruption of cis-interactions releases NK receptors and enhances NK cell functional response. Interestingly, silencing of HS3ST3B in NK cells was found to down-regulate the cis-interactions between HS and the NK receptors KIR2DL4 and NKp46, meaning that the functions of these receptors can be regulated through interactions with 3-*O*-sulfated HS (63, 64). It is thus tempting to speculate that 3-*O*-sulfation in cancer cells may allow cell surface HS to engage in trans interactions with NK cell receptors. Accordingly, upregulation of the expression of certain HS3STs, such as HS3ST3B or HS3ST4, may be a mechanism that permits cancer cells to impact NK cell activation and to escape their elimination. On that assumption, the tumor-promoting properties of HS3STs may rely not only on alteration of intrinsic processes in cancer cells but also on a non-cell autonomous mechanism bypassing immune surveillance.

## Conclusions and Prospects

The regulation of HS biosynthesis is still poorly understood, and whether other factors can influence specific HS sulfation in a given cell type remains largely unknown. In this respect, we demonstrated that HS3ST3B is a Golgi-resident enzyme, while HS3ST2 is specifically addressed to the plasma membrane. This suggests that different subcellular location of HS3STs may be a regulatory mechanism to produce distinct 3-*O*-sulfated motifs (65). Initially, HS3STs have been divided into two groups, based on their contribution to the synthesis of anticoagulant-active sequences and binding motifs for HSV-1 gD protein. However, there is recent evidence that the situation is not so simple, and a better characterization of the catalytic activity of HS3STs and its regulation is required to define more precisely the biological functions of each isozyme (40, 42, 66, 67). It is also of note that most of the effects attributed to HS3STs in cancer are arising from *in vitro* experiments. These findings need to be regarded with caution, because interference with other metabolic processes can have a dramatic impact on cell behavior, without being linked necessarily to changes in HS sulfation (68, 69). All of this suggests that alteration in the expression of HS3STs in cancer cells may have diverse functional impacts, which could explain the different action of a particular isoform in a given cell type. Accordingly, the roles of HS3STs in cancer need to be further explored to evaluate the potential of these enzymes as targets for therapeutic strategies in cancer treatment.

Up to now, a lot of attention has been focused on HS mimetics (6, 70). A typical example is the synthesis of the pentasaccharide that binds antithrombin III (71). However, it is still difficult to synthesize oligosaccharides with complex sulfation patterns. A seducing alternative is the use of a chemo-enzymatic approach, in which controlled sulfation could be achieved by recombinant enzymes (42, 72, 73). To date, most of the HS3STs have been cloned and used to prepare 3-*O*-sulfated oligosaccharides (13, 40, 41, 43). Some of them have proven to be effective as anticoagulant agents (74) and inhibitors of HSV-1 entry (75). On the other hand, targeting HS3STs directly to hinder the reaction of 3-*O*-sulfation may be a challenging endeavor. Byrne et al. (76) reported that HS2ST was a target for a variety of cell-permeable small molecules, including kinase inhibitors. These findings suggest that such molecules could be redesigned for specific inhibition of HS sulfotransferases. On this assumption, designing specific HS3ST inhibitors via high-throughput screening of bio-active agents might be a future strategy to control HS 3-*O*-sulfation in cancer cells. In conclusion, a better understanding of the functions of HS3STs in cancer cells may provide opportunities to use these HS-modifying enzymes as molecular targets to improve therapeutic strategies.

## Author Contributions

AD and FA: conception, design, writing, and review of the manuscript.

### Conflict of Interest Statement

The authors declare that the research was conducted in the absence of any commercial or financial relationships that could be construed as a potential conflict of interest.
